# Measles Vaccination Coverage Survey in Moba, Katanga, Democratic Republic of Congo, 2013: Need to Adapt Routine and Mass Vaccination Campaigns to Reach the Unreached

**DOI:** 10.1371/currents.outbreaks.8a1b00760dfd81481eb42234bd18ced3

**Published:** 2015-02-02

**Authors:** Julita Gil Cuesta, Narcisse Mukembe, Palle Valentiner-Branth, Pawel Stefanoff, Annick Lenglet

**Affiliations:** Infectious Disease Epidemiology, Statens Serum Institute, European Programme for Intervention Epidemiology Training (EPIET), European Centre for Disease Prevention and Control (ECDC), Copenhager, Denmark; Médecins Sans Frontières, Operational Center Amsterdam, Lubumbashi, Democratic Republic of Congo; Department of Infectious Disease Epidemiology, Norwegian Institute of Public Health, Oslo, Norway; Public Health Department, Médecins Sans Frontières, Operational Center Amsterdam, Amsterdam, The Netherlands

**Keywords:** Measles, vaccination

## Abstract

The Democratic Republic of Congo (DRC) has committed to eliminate measles by 2020. In 2013, in response to a large outbreak, Médecins Sans Frontières conducted a mass vaccination campaign (MVC) in Moba, Katanga, DRC. We estimated the measles vaccination coverage for the MVC, the Expanded Programme on Immunization routine measles vaccination (EPI) and assessed reasons for non-vaccination. We conducted a household-based survey among caretakers of children aged 6 months-15 years in Moba from November to December 2013. We used a two-stage-cluster-sampling, where clusters were allocated proportionally to village size and households were randomly selected from each cluster. The questionnaire included demographic variables, vaccination status (card or oral history) during MVC and EPI and reasons for non-vaccination. We estimated the coverage by gender, age and the reasons for non-vaccination and calculated 95% confidence intervals (95% CI). We recruited 4,768 children living in 1,684 households. The MVC coverage by vaccination card and oral history was 87% (95% CI 84-90) and 66% (95% CI 61-70) if documented by card. The EPI coverage was 76% (95% CI 72-81) and 3% (95% CI 1-4) respectively. The MVC coverage was significantly higher among children previously vaccinated during EPI 91% (95% CI 88-93), compared to 74% (95% CI 66-80) among those not previously vaccinated. Six percent (n=317) of children were never vaccinated. The main reason for non-vaccination was family absence 68% (95% CI 58-78). The MVC and EPI measles coverage was insufficient to prevent the recurrence of outbreaks in Moba. Lack of EPI vaccination and lack of accessibility by road were associated with lower MVC coverage. We recommend intensified social mobilization and extended EPI and MVCs to increase the coverage of absent residents and unreached children. Routine and MVCs need to be adapted accordingly to improve coverage in hard-to-reach populations in DRC.

## Background

The World Health Organization (WHO) member states have adopted the goal of measles elimination in five WHO regions, including Africa by 2020 1. Based on the experience of global eradication programmes, the success of a disease elimination depends on the worst-performing country 2. In 2011-2012, the WHO Regional Office for Africa reported the highest number of cases of measles of which 68% were reported by the Democratic Republic of Congo (DRC) 3. One of the targets of measles elimination is vaccinating at least 95% of children in all districts with the first measles dose 4. Priority interventions for the region are improving immunization coverage by strengthening routine and supplementary immunization activities targeting susceptible population groups. The DRC’s Expanded Programme of Immunization routine measles vaccination (EPI) includes one dose of measles vaccine administered to infants aged 9-11 months 5. In 2010, WHO and UNICEF estimated that the EPI measles vaccine coverage among children younger than 12 months was only 68% 6. From 2011, a resurgence of measles cases has been reported from the country, with 133,802 cases in 2011 and 72,029 in 2012 7 ^,^ 8 ^,^ 9

Moba territory is located in Katanga province along Lake Tanganyika and is divided into two Health Zones (HZ), Moba and Kansimba. Moba territory comprises an estimated population of 450,123 inhabitants according to the census. In 2007, Médecins Sans Frontières (MSF) conducted a mass vaccination campaign (MVC) targeting 100,000 children aged from 6 months to 15 years 10 In July 2011, the Ministry of Health implemented a catch up campaign within four days in Moba health zone, with an administrative coverage (number of doses administered divided by the estimated population) of 95% in children aged 6 months to 15 years. From February to November 2013, Moba territory reported 1,500 measles cases with case fatality of 2% and 5% in Moba and Kansimba HZ, respectively. Approximately 80% of cases were children under five years old. In response to the outbreak, from September to December, MSF conducted a MVC targeting 193,360 children aged from 6 months to 15 years. During this campaign 150,354 children were vaccinated. MSF estimated that the administrative vaccination coverage following this campaign was 77% 11.

In order to assess the impact of this MVC, we conducted a survey in Moba territory to estimate the vaccination coverage by age group. The secondary objectives of the survey were to estimate the coverage of measles vaccine administered by the EPI, to identify reasons for non-vaccination, to assess the knowledge of measles among children’s caretakers and to identify the means by which caretakers received information about the measles MVC.

## Methods

**Survey population**

We defined the survey population as children from 6 months to 15 years old and their caretakers living in Moba territory from 13 November to 11 December 2013.

**Definitions**

We defined a respondent as the main adult caretaker of the child included in the survey. We defined a household as a group of people who were under the responsibility of one person, regularly sleeping under the same roof and eating together for at least three months. We defined a measles case as an individual presenting fever and at least one of the following signs: rash, conjunctivitis, runny nose or cough. We defined vaccination verified by card as written documentation of a measles containing vaccine dose recorded during the MVC or EPI. We defined vaccination verified by oral history as the caretaker report of a measles containing vaccine dose, during the MVC or EPI, not confirmed by written documentation.

**Sampling**

We used a two-stage cluster random sampling stratified by HZ. For the first stage, villages were selected using a probability proportional to the size of the villages in each health zone. No villages were excluded from the sampling frame. At the second stage, the first household was randomly selected from the clusters using the modified WHO-EPI method 12. The next selected household was that located closest to the first surveyed household. When a village did not contain the households required to complete the cluster, we combined it with the closest village using the same method to identify the first household.

**Sample size**

The sample size was calculated for each of the two HZ 13 assuming a post-campaign coverage of 80%, choosing an α error of 5%, anticipating a design effect of 4.5, accounting for a 3% non-response and planning for a desired precision of 6%. Seven hundred sixty-four children aged 6-59 months were required. Assuming the average household size of 5 persons 8 , the proportion of children aged 6-59 months being 17%, 0.9 children aged 6-59 months per household, 875 households had to be recruited. This sample size allowed us to assess the EPI vaccination coverage with a precision of 6.5%, a design effect of 4.5 and assumed vaccination coverage of 75%. Considering the logistical constraints and the accessibility to the target health zones, we decided to include 35 clusters of 25 households in each in the two HZ.

**Data collection**

Eight teams of two interviewers collected the data. Each team surveyed one cluster of 25 households per day. If no children meeting the inclusion criteria were identified in a selected household, only information about the household and general knowledge on measles was collected. As a large number of households were empty, the teams selected the nearest alternative household as a replacement.

Caretakers were interviewed using a standardised questionnaire. Its first part collected information about age and gender of household members and the respondents’ knowledge about measles. The second part collected individual information about targeted children in the household, including age and gender, vaccination status for the MVC and EPI and possible reasons for non-vaccination. The questionnaire was printed in French and administered mainly in Swahili although French and other local languages (Tabwua, Luba and Bemba) were also used depending on the language of preference of the respondent.

**Data quality assurance**

We trained surveyors, data entry persons and provincial health supervisors for two days before the data collection. We carried out a pilot survey to pre-test and revise the questionnaire. The field epidemiologist and two provincial health supervisors directly supervised the data collection and checked data to assure data quality daily.

**Data analysis**

The data was entered using Epidata version 3.1 and analysed with Stata version 12 taking into account the cluster survey design, where sampling weights for the health zones were included. We estimated the vaccination coverage (by gender and age) and the frequency of the reasons for non-vaccination with respective 95% confidence intervals (95% CI). We also estimated the mean time between MVC and the survey date, described the means of transport used to access the village for the MVC and estimated the combined vaccine coverage with one vaccine dose received during the MVC or EPI vaccination. We examined the association between the vaccine coverage and means of transport, time since MVC, knowledge about measles disease and between the MVC and the EPI routine vaccination with respective 95% CI and p-values.

**Protection of human subjects**

The health authorities of Moba and Kansimba HZ and the chief in each village authorized the survey. We requested oral consent from each family before administration of the questionnaire. We vaccinated children if caretakers asked for the vaccine and it was available. The questionnaire was processed anonymously. As this survey was part of the regular monitoring and evaluation activities following MVCs, the approval of the Ethical Review Board of MSF was not required. Raw data is available upon request on http://fieldresearch.msf.org/msf/handle/10144/306488

## Results

We included 68 clusters out of the 70 planned, with a mean of 25 households per cluster. Two clusters were dropped because of security concerns. We interviewed 1,700 household caretakers. Four households (0.2%) did not consent to participate and 12 (0.7%) were excluded because the respondent was less than 18 years old. We included 1,684 households in the analysis (median number of persons per household: 5 [Interquartile range = 2]). Of these, 1,536 (91%) had at least one eligible child for inclusion in the survey.

**Characteristics of the respondents and surveyed children**

The survey respondent was the mother for 62% (n=1,034, 95% CI 59-66) of households and the father for 29% (n=487, 95% CI 27-32) of the households. The mean age of the respondents was 36 years (95% CI 35-36). We surveyed 4,768 children (mean age: 6.6 years (95% CI 6.4-6.8); 48% male). Thirty seven percent were aged 6-59 months, 37% were aged 5-10 years and 26% were aged 10-15 years.

**Vaccination coverage of the MVC, 2013**

Information on vaccination status was available for 4,766 children (99%). Vaccination coverage was 65% (95% CI 61-70) as documented by the vaccination card and 87% (95% CI 83-90) as determined by both card and oral history. Vaccination coverage did not vary by age group or gender (Table 1).

Of the 68 villages, the MVC accessed 45 villages by car and 23 villages (not accessible by car) using boat, motorbike or foot. The coverage in villages accessed by car was 89% (95% CI 85-92), higher than in those not accessible by car where it was 82% (95% CI 75-87, p-value < 0.05).

**Figure:**
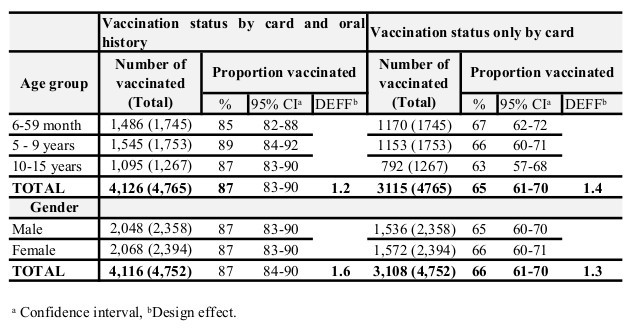
**Table 1. Measles mass vaccination campaign coverage: by age group and gender in Moba, Katanga, DRC, 2013.**

**EPI vaccination coverage**

Information on the EPI measles vaccination at 9 months of age was available for 4,758 (99%) children. Vaccination coverage was 3% (95% CI 2-4) as documented by health centre immunization card and 76% (95% CI 71-81) as documented by immunization cards and oral history. Vaccination coverage did not vary by age group or gender (Table 2).

**Figure:**
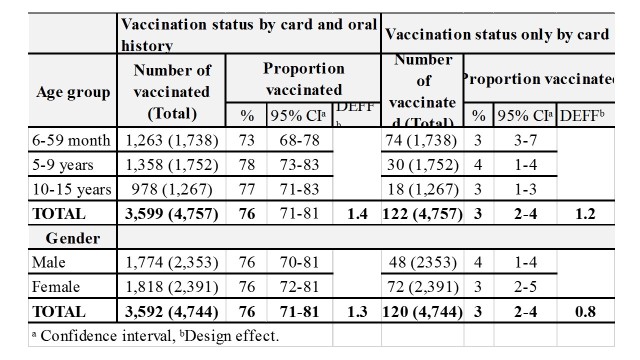
**Table 2. Routine EPI vaccination coverage: by age group and gender in Moba, Katanga, DRC, 2013.**

**Combined MVC and EPI vaccination coverage**

The vaccination coverage was 94% (95% CI 91-96) for doses of measles vaccine received either through EPI or MVC. Of those, 18% (n=841) did not receive EPI vaccine, but reported vaccination during the MVC. Sixty-nine percent (n=3,278; 95% CI 64-74) of children received both MVC and EPI doses as determined by oral history. Six percent (n=317; 95% CI 4-9) of children had not benefited from any of the two immunization opportunities. The coverage for the MVC was significantly higher among children previously vaccinated during EPI 91% (95% CI 88-93), than among others 74% (95% CI 65-80) (p-value < 0.001).

**Reasons for non-vaccination**

Children who had not been vaccinated for any of the campaigns reported that absence of the family in the village on the vaccination day was the main reason for non-vaccination, when they were asked about MVC (33%) and EPI (71%) (Figure 1). The second most reported reason among these not vaccinated children was lack of knowledge about time or place of vaccination during the MVC (5%) and EPI (28%). The vaccination site being too far away was also mentioned by these children for the MVC (5%) and EPI (10%) (Figure 1).

**Reasons for non-vaccination according to vaccination status in Moba territory, DRC, 2013.**
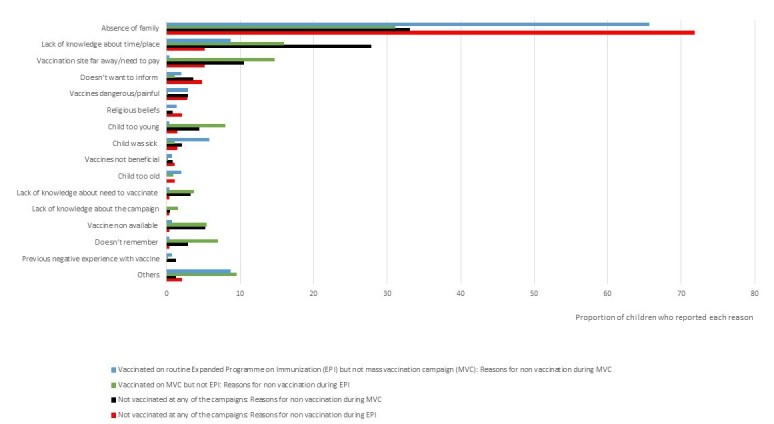

**Knowledge about measles disease and vaccine**

Ninety-one percent of respondents (n=1,524) knew about measles. Sixty percent of respondents knew the symptoms (63% named fever and 73% red eyes). MVC vaccination coverage was not associated with respondents’ knowledge about measles. Seventy-nine percent of respondents (n=1,324) knew about measles vaccine. MVC vaccination coverage was higher in children whose caretakers knew about the vaccine 88% (95% CI 85-91) than among others 76% (95% CI 64-85).

**Means of knowledge about the MVC campaign and time from MVC to survey**

Respondents stated that they had heard about the MVC through social mobilizers 67% (95% CI 60-74), who communicated door to door and/or through megaphones. The information was also received through the radio 26% (95% CI 20-32) where available.

Nineteen percent of children had been vaccinated in the week before the survey, 40% in the preceding 7-30 days and 41% more than one month before. The MVC coverage as documented by card was higher among children who were vaccinated in the week before the survey 84% (95% CI 77-90), than among those vaccinated in the previous month 60% (95% CI 52-68), and among those vaccinated more than 30 days before 62% (95% CI 56-68) ( p value < 0.001).

## Discussion

The measles vaccination campaign in Moba territory in DRC in 2013 did not achieve the 95% coverage that is the target for vaccination campaigns to eliminate measles 4 ^,^ 8. Our coverage estimates contrast with the estimated administrative coverage of the campaign (77%) underlining the importance of field-based coverage surveys 14 for reliable estimates.

We identified three main barriers to vaccination during the MVC: accessibility of the villages for vaccination teams, lack of a EPI measles dose at nine months and the absence of families at the time of vaccination. All three factors are likely to be connected and illustrate how intrinsic characteristics of the target communities may influence coverage and not necessarily how the vaccination campaign is implemented or by whom.

First, vaccination coverage was higher in villages accessible by car compared to those not accessible. In DRC, the population lives across large geographic areas with very limited road and public transport networks. Logistical challenges of accessing children in distant areas are overwhelming and constant 14 ^,^ 15 .

Second, 69% of children had received one dose of vaccine during the MVC campaign and another one through the EPI. This illustrated that repeated supplementary immunization activities may target the same children and systematically miss the same population subgroups 16. Hence, understanding why 6% (n=317) of children surveyed had never been vaccinated is crucial. The hard-to-reach populations in DRC and similar settings must be provided with additional resources to cope with logistical, transport, cold chain and human resource limitations 7 ^,^ 15 .

Third, with respect to the absence of the family during the vaccination day, we anecdotally noted that families farming in distant areas may have played a role. During the MVC, the majority of families were informed about the vaccination campaign through social mobilizers. Sensitization through social mobilizers is a useful and effective communication tool 17 in reaching and encouraging families to bring their children for vaccination. However, the coverage reached after the campaign suggests that it must be improved. The use of religious 18 and community leaders in addition to social mobilizers for further social mobilization should also be considered in future MVCs, especially in villages with lower EPI coverage. Villages require information well in advance about the vaccination location and time. The time spent at vaccination sites should also be extended in less accessible villages to ensure that absent residents have time both to learn about the vaccination and return from the farming fields to ensure vaccination in future MVC and routine EPI vaccination campaigns.

Despite the short time between the MVC and the coverage survey, the difference between the coverage documented by MVC immunization card and oral history was 22%. Factors that influenced retention of the vaccination cards were not addressed in the survey. However, we anecdotally observed how children were playing with their card and how families reported that cards were kept by different family members. In future campaigns, improved card retention would allow more homogenous coverage estimates regardless of the time of the survey. It would ensure that vaccination coverage can be rapidly and accurately determined 19. This may help identifying children not reached by any of the previous campaigns 18.

The main limitation of the survey was the high number of empty houses in the surveyed villages (anecdotally up to 30% in some villages). This was due to absence of farmers working in distant areas, according to village chiefs and neighbours. This could lead to selection bias: non-surveyed empty houses may have also been empty during the MVC and subsequently not offered vaccination. Furthermore, as the coverage by oral history is based on self-reporting, recall bias may have affected the estimated coverage for the MVC and the EPI 18. For the MVC, we minimized this bias by the short time between the MVC and the survey. The EPI coverage estimate should be cautiously interpreted however, as some respondents may have misunderstood which vaccine the interviewers asked about. To increase the accuracy of responses, we used the local term for measles during the interviews 20 and asked parents to describe the part of the body where the vaccine was delivered to check whether they distinguished measles from other vaccines. Even so, previous studies show that in areas of high measles incidence there is also a high reliability of parents’ recall about the vaccination status of their children 21.

## Conclusions

We estimated 87% coverage of the MVC in response to the measles outbreak in Moba territory. This coverage may be insufficient to prevent future outbreaks. Lack of a EPI vaccination and lack of accessibility by road were associated with lower MVC campaign coverage. Absence during the MVC and EPI vaccination were the main reasons for non-vaccination. On the basis of these conclusions, we recommend more accessible vaccination sites for each village in order to improve vaccination coverage during EPI and MVCs. We recommend improved social mobilization of the population through extended vaccination time in less accessible villages and to give notice well ahead of vaccination days. Campaign staff must emphasise children and their parents the importance of keeping the vaccination cards. EPI and MVCs need to be adapted accordingly to face these logistical and communication barriers. Hence, the vaccination of hard-to-reach children can contribute to meet the goal of measles elimination in DRC and similar settings.
